# Comparative Analysis of Volatiles of 15 Brands of Extra-Virgin Olive Oils Using Solid-Phase Micro-Extraction and Solvent-Assisted Flavor Evaporation

**DOI:** 10.3390/molecules24081512

**Published:** 2019-04-17

**Authors:** Qi Zhou, Shaomin Liu, Ye Liu, Huanlu Song

**Affiliations:** 1 Beijing Engineering and Technology Research Center of Food Additives, Beijing Advanced Innovation Center for Food Nutrition and Human Health, School of Food and Chemical Engineering, Beijing Technology and Business University (BTBU), Beijing 100048, China; zhouqi01@caas.cn (Q.Z.); liushaomin2017@sina.com (S.L.); caucfsne@gmail.com (H.S.); 2 Oil Crops Research Institute of the Chinese Academy of Agricultural Sciences, Oil Crops and Lipids Process Technology National& Local Joint Engineering Laboratory, Wuhan 430062, Hubei, China

**Keywords:** extra-virgin olive oils (EVOOs), headspace solid-phase micro-extraction (SPME), solvent-assisted flavor evaporation (SAFE), cluster analysis, quantification

## Abstract

Aroma profiles, key aroma compound quantification, and cluster analysis of 15 brands of extra-virgin olive oils (EVOOs) from three countries (Spain, Italy, and Greece) were investigated in the current study. Aroma compounds were isolated from the oil by using solvent-assisted flavor evaporation (SAFE) and solid-phase micro-extraction (SPME) and analyzed by gas chromatography-olfactometry mass spectrometry (GC-MS/O). A total of 89 compounds were screened by SPME/SAFE-GC-MS/O with chromatographic columns in 15 brands of samples. Eighty and 54 compounds were respectively identified by SPME- and SAFE-GC-MS/O. Of those, 44 compounds were detected by both methods. Undecanol, (Z)-4-decenal, (E)-2-dodecenal, and 2-nonanone extracted by SAFE were not found in EVOOs before. Eight classes of aroma compounds were identified, including 17 alcohols, 22 aldehydes, 9 ketones, 4 acids, 14 esters, 5 aromatics, 12 alkene, and 6 others. Eleven compounds were identified as the key aroma compounds in alternative brands of EVOOs by SAFE-aroma extract dilution analysis (AEDA). Hexanal, (E)-2-hexenal, (E)-3-hexenol, acetic acid, and (E)-2-heptenal were the common key aroma compounds by AEDA and odor activity values (OAVs). From the cluster analysis of the heatmap, the aroma compounds of all the Spain EVOOs were similar, and there were some differences from the samples of Italy and Greece. It suggested that both the amount and concentration of aroma compounds determine the similarity of aroma in EVOOs.

## 1. Introduction

Extra-virgin olive oil (EVOO) is a kind of high-grade edible vegetable oil obtained by cold mechanical extraction from fresh olives (*Olea europaea*) without use of solvents or refining methods [1]. It can be consumed in original form without refining, possessing good stability as well as nutritional and healthy features with respect to other vegetable oils [2]. Thanks to its excellent nutritional and organoleptic properties, EVOO has become one of the most popular oils in the world. The demand for it has increased rapidly in the past decade. In 2014, 3.05 million tons of olive oil was produced worldwide, and the production of olives increased to 19.27 million tons in 2016 [3].

Aroma is an important criterion for EVOO. Its characteristic flavor is one of the main f distinguishing it from the other edible vegetable oils [4]. Meanwhile, the identification of the compounds contributing to the aroma is considered to be a key for quality and authentication control. The aroma compounds of EVOO derive from the enzymatic reactions and autoxidation of unsaturated fatty acids; they occur by the lipoxygenase (LOX) pathway comprising mainly the actuation of LOX and hydroperoxide-lyase enzymes [5] after crushing and during malaxation, a basic step of EVOO extraction process, aimed to improve oil yield and quality [6,7]. From the qualitative and quantitative results, C6 aldehydes and alcohols and the corresponding esters are considered to be the most crucial aroma compounds of EVOO [8,9]. Reasonable amounts of various classes of C5 compounds are also contained in the aroma of EVOO [10].

Aroma of EVOO consists of a complex mixture of volatile compounds, which includes mainly aldehydes, alcohol, ketones, and esters [2,11,12]. (*E*)-2-Hexenal is the most important compound in olive oil, followed by hexanal and (*E*)-2-heptenal using the extraction methods of headspace SPME, purge and trap (P&T), simultaneous distillation and extraction (SDE), and headspace (HS) [2,11,13,14]. According to the flavor dilution (FD) factor, the most potent aromatic active compound is hexanal (FD = 512) in Fakhari olive oil, (FD = 256) in Touffehi oils, and (FD = 128) in Jemri olive oil [12]. (*E*)-2-Hexenal, (*E*)-2-hex-1-enol, (*Z*)-3-hexen-1-ol, and 1-hexanol were also identified in the virgin olive oils from Greece and Tunisia of Koroneiki variety using SPME-GC/MS [8]. Although the percentage of C6 alcohols [hexan-1-ol, (E)-2-hexen-1-ol, and (E)-3-hexen-1-ol] were higher than that of C6 aldehydes [hexanal and (E)-2-hexenal], C6 aldehydes contributed to the virgin olive oil from four cultivars greatly grown in southern of Tunisia with the lower thresholds [15]. C6 and C5 volatile compounds were considered to be the potent odorants in most studies previously, but there were some differences of varieties and content of the compounds because of the different extraction methods [2,11,13,14,16].

The selective adsorption coating of SPME results in the incomplete extraction of aroma compounds. The high temperature of SDE affects the accuracy during the extraction process. Solvent-assisted flavor evaporation (SAFE) has become a preferred method because of the low extraction temperature and high vacuum characteristics (about 10^−5^ Pa), which can extract more low-boiling compounds. SAFE allows the fast and careful isolation of volatiles from either solvent extracts of foods, oil samples, or even fruit pulps. Application of SAFE to model solutions of selected aroma compounds resulted in higher yield from both solvent extracts and fatty matrices (50% fat) compared to previously used techniques. Besides its efficiency in aroma isolation, the use of the equipment saves time and reduces costs due to the stability of the compact distillation unit [17]. The volatile profiles of pomelo flower, leaf, peel, and juice were comparatively analyzed using HS-SPME and SAFE [18]. To our knowledge, SAFE has not been applied to the aroma extraction of EVOO until now. In addition, the difference among EVOO brands from different countries was not evaluated previously. From this viewpoint, in this study, (1) SAFE coupled with aroma extract dilution analysis (AEDA) was applied to identify the key aroma compounds of 15 brands of EVOO from Spain, Italy, and Greece; (2) the most contributing compounds were quantified by standard curve method using selective ion monitoring (SIM) mode by SPME; (3) the EVOOs were classified based on their volatile compounds.

## 2. Results and Discussion

### 2.1. Aroma Compounds of 15 Brands of EVOOs Extracted by SPME and SAFE

Eighty-nine compounds were screened by SPME/SAFE-GC-MS/O with chromatographic columns of DB-Wax and DB-5 (Table 1). Eight classes of flavor compounds were identified in the 15 brands of EVOOs, including 17 alcohols, 22 aldehydes, 9 ketones, 4 acids, 14 esters, 5 aromatics, 12 alkene, 6 others. The important aroma compounds in EVOOs are the C6 compounds, which were generated from lipoxygenase pathway through a series of enzymatic acting on fatty acids, such as linoleic and linolenic acids initiated by the tissue disruption [5]. The hydroperoxide-lyase enzyme produces aldehydes, subsequently reduced to alcohols by the alcohol dehydrogenase enzyme [19].

#### 2.1.1. Alcohols

In this study, 17 alcohols were identified from the 15 brands of EVOOs (Table 1). Among them, hexanol, (*E*)-3-hexenol, (*Z*)-3-hexenol, and phenylethyl alcohol existed in all the 15 EVOOs. Compared to previous studies, these four compounds were also detected in virgin olive oils produced in Iran, Turkey, Spain, Greece, Tunisia, and Italy [2,8,11,12,15,16,20,21,22,23]. (*E*)-3-Hexenol and (*Z*)-3-hexenol are derived from the reduction of (*E*)-3-hexenal and (*Z*)-3-hexenal by the action of an alcohol dehydrogenase (ADH). They present the most obvious green, fresh, and grass note. However, these aroma compounds were not thought to be responsible for the significant effect on olive oil odor, owning to their high odor threshold values in oil [19,24]. 2-Ethyl hexanol, linalool, octanol, 1-nonanol, undecanol, and benzyl alcohol were found in most of the 15 brands of EVOOs. 2-Ethyl hexanol, octanol, 1-nonanol, and benzyl alcohol were also reported in the previous studies about EVOOs from Turkey, Spain, Greece, Tunisia [2,8,16,23]. Nonanol presents fat or green note, which also derives from lipoxygenase pathway of unsaturated fatty acids [19]. Linalool with the flower and lavender note was detected in the samples of Turkey [2], while undecanol was not found in previous studies on EVOO flavor.

#### 2.1.2. Aldehydes

In this study, 22 aldehydes were identified from the 15 brands of EVOOs (Table 1). Among them, hexanal, (*E*)-2-hexenal, octanal, nonanal, (*E,E*)-2,4-heptadienal, and (*E*)-2-decenal were detected in all the 15 brands of EVOOs. Also, these compounds were reported in the previous studies on EVOOs from Iran, Turkey, Greece, Tunisia, and Italy [2,8,9,11,15,23]. The enzymatic breakdown of the 13-hydroperoxide of linolenic acid in leaf homogenates causes to produce the (*E*)-2-hexenal, while the action of the aldehyde-lyase generates hexanal [25]. Hexanal, (*E*)-2-hexenal, octanal, and nonanal present grass or green note, while (*E,E*)-2,4-heptadienal and (*E*)-2-decenal show the fat or tallow note. (*E*)-2-Heptenal, (*E*)-2-octenal, benzaldehyde, (*E*)-2-nonenal, and (*E,E*)-2,4-decadienal were existed in most of the 15 brands of EVOOs. These aroma compounds also found in the EVOOs from Turkey and Spain [2,16,23]. (*E*)-2-Heptenal, (*E*)-2-octenal, (*E*)-2-nonenal, and (*E,E*)-2,4-decadienal all possess the fat note in their flavor quality, and benzaldehyde showed almond or burnt sugar note. While the other compounds, for example (*E*)-2-pentenal, (*Z*)-2-hexenal, 2-furanaldehyde, and phenylacetaldehyde, only existed in some brands of EVOOs, most of the aldehydes have the low odor threshold values in common, which contribute to the whole flavor of EVOOs greatly. However, hexanal, (*E*)-2-hexenal, octanal, nonanal, (*E,E*)-2,4-heptadienal, and (*E*)-2-decenal were also reported as being potentially associated with off-flavor sensations and organoleptic defects in their elevated concentrations, such as winey, fusty, frozen, musty, or hay-wood as in note [26,27]. In the next research, the panel test will be used to verify if the 15 brands of EVOOs present olfactory defects due to the compounds above, because the perceived intensity of the defect can compromise the quality of the oils and determine a downgrading based on the law.

#### 2.1.3. Ketones

In this study, 9 ketones were identified from the 15 brands of EVOOs (Table 1). Among them, 1-octen-3-one and 6-methyl-5-hepten-2-one, both with the mushroom note, were found in all the 15 brands of EVOOs. In the previous studies, 6-methyl-5-hepten-2-one was also reported in the virgin olive oil samples from Iran, Turkey, Spain, Tunisia [2,11,12,15,16,20,23,27]. While 1-octen-3-one was only detected in 1 kind of Turkish olive oils until now [22], 3-Pentanone and 3,5-octadien-2-one existed in the 15 brands of EVOOs comprehensively. 3-Pentanone showing ether note was also found in the samples of Spain, Greece, Tunisia [7,15,19,22,28], whereas 3,5-octadien-2-one with fruit, fat, and mushroom note only appeared in the 3 kinds of Turkish olive oils in the previous investigations [23]. 1-Penten-3-one, 2-octanone, 2-nonanone, and acetophenone existed in few samples of the 15 brands of EVOOs.

#### 2.1.4. Acids

In this study, 4 acids were identified from the 15 brands of EVOOs, including acetic acid, propionic acid, hexanoic acid, and nonanoic acid (Table 1). These 4 acid compounds were not detected in all the 15 brands of EVOOs. They were all found in the study on Turkish olive oils as well [23]. Many kinds of virgin olive oils did not contain acid compounds [8,11,15,16,20,22,28]. Acetic acid was reported in the olive oil samples of Turkey, Tunisian [2,12,29]. Propionic acid and hexanoic acid existed in Iranian olive oil [11]. Nonanoic acid and acetic acid were related to fusty and winey-vinegary defects, respectively [26]. Negative aromas such as rancid, fusty, winey-vinegary, and frozen are sensory attributes of defective virgin olive oil recognized by the International Olive Council. In the next research, the panel test will also be applied to confirm if the 15 brands of EVOOs show olfactory defects due to nonanoic acid and acetic acid, as the oil quality will be compromised by the perceived intensity of the defect and lead to a downgrading.

#### 2.1.5. Esters

In this study, 14 ketones were identified from the 15 brands of EVOOs (Table 1). Among these compounds, hexyl acetate, (*Z*)-3-hexenyl acetate, methyl benzoate, and methyl salicylate were found in all the 15 brands of EVOOs. They were also detected in the virgin olive oil from Italy, Greece, and Tunisia [8,9]. Besides, hexyl acetate and (*Z*)-3-hexenyl acetate were reported in the sample of Portugal, Turkey, Tunisia, and Spain, which possess the fruit and green note [2,12,13,15,20,22,23]. Methyl benzoate in the samples of Iran and Spain, and methyl salicylate in the ones of Tunisia and Turkey presented the herb and peppermint note [11,12,16,23]. Ethyl benzoate existed in several kinds of EVOOs with chamomile or flower note, which was reported in the samples from Iran [11].

#### 2.1.6. Aromatics

In this study, 5 aromatics were identified from the 15 brands of EVOOs (Table 1). Thereinto, methylbenzene, 1,2-dimethylbenzene, 1,3-dimethylbenzene, and 1,2,4-trimethylbenzene were detected in most of the samples. In previous study, 1,2-dimethylbenzene and 1,3-dimethylbenzene with geranium and plastic note existed in the samples from Greece and Tunisia [8], and 1,2,4-trimethylbenzene with plastic note was found in the virgin olive oil from Spain and Italy as well [9,16,20,28]. Methylbenzene and 1,2,4,5-tetramethylbenzene with the paint and rancid note were not reported in the former investigations.

#### 2.1.7. Terpenes

In this study, 12 terpenes were identified from the 15 brands of EVOOs (Table 1). (-)-α-Copaene and α-farnesene existed in all the 15 brands of EVOOs with the wood, spice, and sweet note. (-)-α-Copaene was reported in the virgin olive oil from Turkey, Spain, Greece, Tunisia, and Italy [2,8,9,16,23], and α-farnesene was found in the samples of Iran, Spain, Tunisia, and Italy as well [9,11,12,16,30]. (+)-Dipentene, 3-carene, (*Z*)-3,7-dimethyl-1,3,6-octatriene, and ethynylbenzene were detected in most of the 15 brands of EVOOs. 3-Carene with the lemon and resin note was also reported in the virgin olive oil of Spain [16,20]. While (*E*)-ocimene was only detected in the sample, OS were found in several kinds of olive oil from Spain, Tunisia, and Turkey [12,20,23].

#### 2.1.8. Others

In this study, 6 other compounds were identified from the 15 brands of EVOOs (Table 1). All these compounds (2-pentylfuran, benzyl methyl ether, dimethyl sulfoxide, naphthalene, 4-ethylphenol and 4-allylanisole) only existed in some brands of samples. They had no contribution to the aroma of all the samples by FDs. Based on the studies before, there was no report about the compounds above in EVOOs. These six compounds were not the important aroma compounds in EVOOs.

### 2.2. Comparison of Extraction Effect of SPME and SAFE

**Figure 1:**
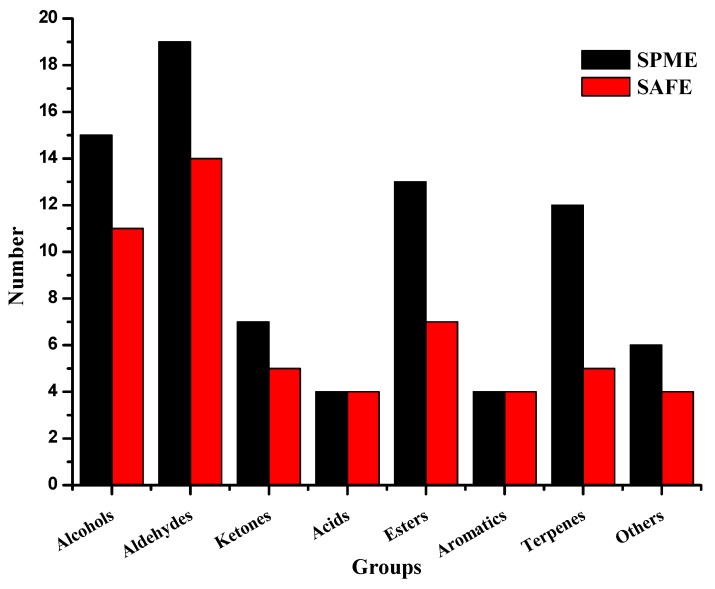
Number of different groups of flavor compounds extracted by SPME and SAFE.

Eighty compounds in EVOOs were identified by SPME-GC-MS/O and 54 by SAFE-GC-MS/O method (Table 1). Forty-four compounds were detected by both methods. Two extraction methods (SPME and SAFE) had different extraction efficiency for different kinds of volatile compounds (Figure 1).

It can be seen from Figure 1 that more alcohols, aldehydes, ketones, esters, terpenes, and others were detected by SPME compared to SAFE. The same numbers of acids and aromatics were extracted by these two methods. The reasons can be the following: formyl groups of aldehydes and carbonyl groups of ketones were unstable, which could get oxidized or reduced in organic solvent. Alcohols in EVOOs are derived from the reduction of aldehydes by dehydrogenase, for example, from (*E*)-2-pentenal to 1-pentene-3-ol, from (*E*)-2-hexenal to (*E*)-2-hexenol, from octanal to octanol, which resulted in the more alcohols by SPME [31]. This result was in accordance with that of watermelon juice [32]. It indicated that SPME had better extraction effect on aldehydes, ketones, and alcohols just because of the different systems of EVOOs and watermelon juice.

Similarly, SPME could extract more esters than that by SAFE, while the result was just opposite in natto [33]. In natto, all the esters were ethyl esters, and there were more complicated constitutes of esters in EVOOs including ethyl, methyl, hexyl, and linalyl ester, which might result in the different extraction effect. SPME also had better extraction effect to terpenes. The previous studies showed that SPME with the fiber of DVB/CAR/PDMS (divinylbenzene/carbon/polydimethylsiloxane) had better extraction effect to terpenes compared to the other fibers (100 μm PDMS, 85 μm PA (polyacrylate), and 7 μm PDMS) or method (hydrodistillation), which confirmed the result in this study to some extent [34,35]. In conclusion, SPME had better extraction efficiency to the aroma compounds in EVOOs.

However, nine compounds were only extracted by SAFE, including (*E*)-3-hexenol, undecanol, octanal, (*Z*)-4-decenal, (*E*)-2-dodecenal, 1-octen-3-one, 2-nonanone, butyl acetate, and 1,2,4-trimethylbenzene. In particular, undecanol (mandarin), (*Z*)-4-decenal (green, must), (*E*)-2-dodecenal (green, fat, sweet), and 2-nonanone (hot milk, soap) were found in EVOOs for the first time to the best of our knowledge. All these compounds had a significant aroma property, and 2-nonanone was the key aroma compounds identified by AEDA. (*E*)-3-Hexenol and 1-octen-3-one reported in the previous studies were also the potent aroma compounds in this study.

### 2.3. Identification and Quantification of Key Aroma Compounds of 15 Brands of EVOOs

Eleven compounds were identified as the key aroma compounds in alternative brands of EVOOs by SAFE-AEDA (Table 2). FD value reflects the flavor contribution degree of each compound. Higher value indicates greater contribution to EVOO flavor. Among them, four compounds [hexanal (FD: 16 to 64), (*E*)-2-hexenal (FD: 2 to 64), 1-octen-3-one (FD: 4 to 32), and (*E*)-3-hexenol (FD: 0 to 8)] were the joint key aroma compound in the 15 brands of EVOOs, and the former two compounds contributed to the flavor greatly with the highest FD values, which was in accordance with the low odor thresholds of aldehydes. In particular, (*E*)-2-hexenal in all the Spain EVOOs had high FD (64 in PL, BDS, OL, and YGY/16 in BLN), suggesting its great contribution to Spain samples. The flavor character of hexanal and (*E*)-2-hexenal were grass, fat, apple, or green note represented the flavor of EVOOs to a great extent. In addition, the mushroom/metal note of 1-octen-3-one and moss/fresh note of (*E*)-3-hexenol also impacted the whole flavor of EVOOs significantly. The other three kinds of compounds, 2-octanone (FD: 0 to 16, does not exist in BLN, soap or gasoline note), (*E*)-2-heptenal (FD: 2 to 16, does not exist in PL, fat or almond note), and (*E*)-2-Nonenal (FD: 0 to 16, does not exist in BDS, cucumber and green note) affected the EVOO flavor notably. These three brands of EVOOs were both from Spain. Nonanal (FD: 0 to 8) and 2-nonanone (FD: 0 to 4) existed in most of Italy and Greece EVOOs, but they both did not consist in the Spain samples. It indicated that the EVOOs from Spain were lack of the flavor of fat, citrus, green, milk, or soap. Acetic acid (FD: 0 to 4, 16 in ALCF) and methyl benzoate (FD: 2 to 4, 8 in YGY) only existed in several brands of EVOOs from these three countries, and they had little effect to the flavor of EVOOs.

Among these key aroma compounds identified by SAFE-AEDA, 1-octen-3-one could not be quantified because it had no peak in total ion chromatogram. 2-Octanone could be detected only in four samples by SAFE, so it could not be quantified by SPME with external standard; nonanal, 2-nonanone, (*E*)-2-nonenal and methyl benzoate could only be detected in several samples with the weak contribution (lower FD). Therefore, they were not quantified precisely. Except for the compounds above, all the other five compounds were quantified by external standard. Besides, (*Z*)-3-hexenyl acetate and 6-methyl-5-hepten-2-one were also identified as the key aroma compounds by SPME with internal standard, although they could not be sniffed by SAFE. Hence, all these seven compounds were quantified precisely (Table 3).

As shown in Table 4, (Z)-3-hexenyl acetate showed the highest odor activity value (OAV), indicating the strong contribution to the flavor of EVOOs. (*E*)-2-heptenal, hexanal, acetic acid, and (*E*)-2-hexenal were also identified as the key aroma compounds based on OAVs by SPME. It was in accordance with the results by SAFE-AEDA. While (E)-3-hexenol and 6-methyl-5-hepten-2-one had little aroma contribution due to the lower OAVs, there were some differences between the results based on AEDA and OAV. Hence, the key aroma compounds could be identified precisely by both methods.

### 2.4. Comparison of Aroma Compounds of 15 Brands of EVOOs

As shown in Table 5, the detection frequency of compounds was compared based on the kinds of EVOOs and producing areas. The detection frequency of alcohols in EVOOs of Italy (subtotal: 54) and Greece (subtotal: 58) were higher than that of Spain (subtotal: 39) generally. In particular, there were only five and seven kinds of alcohols in PL and BDS. There was a big difference on that of esters among the samples from these three countries, EVOOs of Spain showed the highest detection frequency (39). Similarly, for the other compounds, there was also the highest detection frequency from samples of Spain. While the ones of aldehydes, ketones, acids, aromatics, and terpenes were very close among the samples of these three areas, for the brand of sample, the range of detection frequency was from 41 to 59. The highest one (OL: 59) and the lowest one (BLN: 41) were both from Spain. The impact factor of aroma compound of EVOOs included the cultivar of olive, growing environment, producing technology, storage condition, and so on. From this aspect, these factors might impact EVOO aroma of Spain greatly.

**Figure 2:**
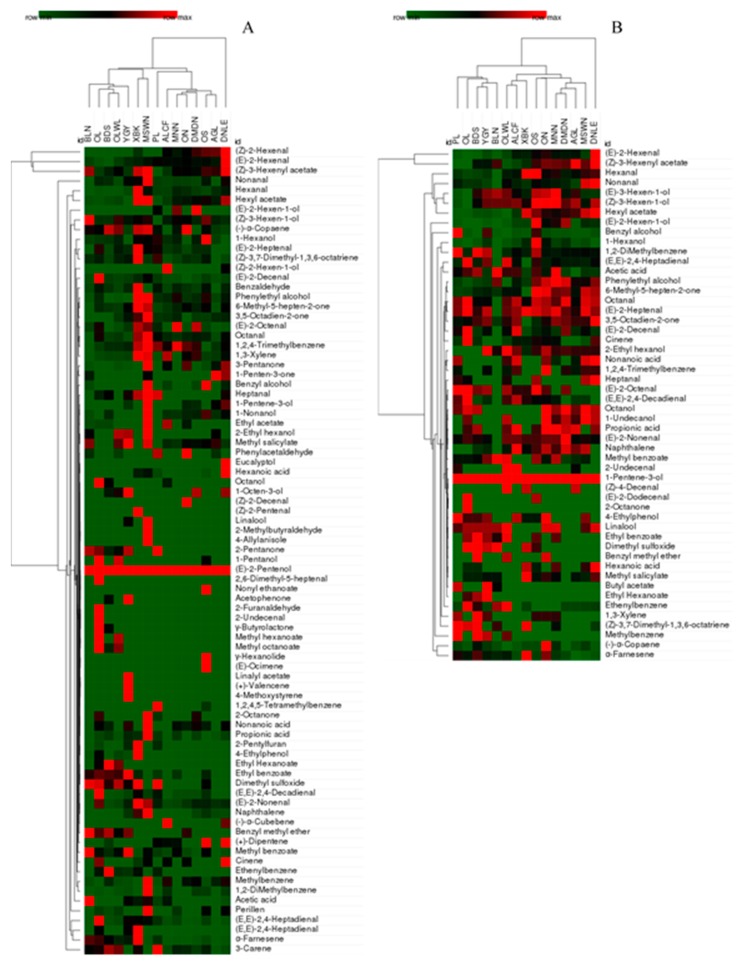
Heatmap indicating the aroma compounds from 15 EVOOs by SPME (**A**) and SAFE (**B**).

Grouping different types of oil is meaningful by flavor because it is an important criterion for EVOO. In the previous studies, cluster analyses were applied to distinguish the different types of edible oil based on their flavor. Sesame oils, soybean oils, and peanut oils could be completely classified using cluster analysis of volatiles [36]. Also, hierarchical cluster analysis showed similarities between EVOO, VOO and LOO (lampante olive oil) samples based on HS-GC-IMS fingerprints [37]. It can be seen from Figure 2, EVOOs were clustered in the heatmap based on their aroma compounds extracted by SPME and SAFE. From Figure 2A, EVOOs of BLN, OL, BDS, OLWL, and YGY had the similar aroma components, all these samples were from Spain except for OLWL. In addition, the flavor was similar between the samples from Italy (ALCF, MNN, AN, and OS) and Greece (DMDN and AGL). From Figure 2B, the aroma compounds of five brands of EVOOs from Spain (PL, OL, BDS, YGY, and BLN) were alike, and they were analogous between the ones from Italy (OS, AN, and MNN) and Greece (DMDN, AGL, and MSWN). Based on these results by SPME and SAFE, the flavor of Spain EVOOs was similar, and that of some of samples from Italy and Greece was alike. It indicated that there was big difference between samples of Spain and the other countries. These results seemed to contradict with that of detection frequency, but it just suggested that the similarity depended not only on the number of compounds but also on the content of ones.

## 3. Materials and Methods

### 3.1. Samples

Fifteen varieties of EVOOs from the three largest export countries of olive oil in the world were purchased from the import supermarket. All the samples were authenticated by “Inspection and Quarantine Certificate of Entry Goods” from Entry-exit Inspection and Quarantine Bureau of the People’s Republic of China. EVOO samples were stored at 15 °C in dark. Specific brands were used in this study as follows, Spain: PL (MUELOLIVA), BDS (BETIS), OL (EURO GOLD), BLN (BELLINA), and YGY (IGAURIN); Italy: OLWL (OLIVOILÀ), OS (OUSA), ALCF (EMROW KITCHEN), MNN (MONINI), and AN (OLITALIA); Greece: XBK (HIPPOCRATES), DMDN (DIAMANDINO), MSWN (MESA VOUNOS), AGL (AEGLE), and DNLE (DONLIAR). All the EVOOs were imported with original packaging from the three countries mentioned above. The quality of all these samples complied with the “Trade Standard Applying to Olive Oils and Olive-Pomace Oils” (COI/T. 15/NC No. 3/Rev. 7 2013) by International Olive Council.

### 3.2. Chemicals

Dichloromethane, anhydrous sodium sulfate, n-hexane were purchased from Yifengtiancheng Scientific Instruments Co. Ltd. (Beijing, China); n-Alkanes (C7~C30) and 4-methyl-2-pentanol were purchased from Sigma-Aldrich (St Louis, MO, USA); Nitrogen (99.9992% purity) was purchased from Beijing Haipu Beifen Gas Industry Co. Ltd. (Beijing, China).

Standards of volatile compounds, hexanal, trans-2-hexenal, (*Z*)-3-hexenyl acetate, trans-2-heptenal, 6-methyl-5-hepten-2-one, (*Z*)-3-hexen-1-ol, acetic acid, 2-octanone, and 1-octen-3-one were purchased from Sigma-Aldrich Co. Ltd. (Santa Clara, CA, USA).

### 3.3. Aroma Extraction of EVOO by SPME

The method was in accordance with Wang et al. with minor modification [38]. After being selected, a manual SPME (Supelco, Inc., Bellefonte, PA, USA) with a 50/30 μm divinylbenzene/carboxen/polydimethylsiloxane (DVB/CAR/PDMS) SPME fiber was used for volatile extraction after the fiber had been conditioned at 250 °C for 30 min. Ten milliliter of EVOO sample was quickly transferred into a 40 mL vial, 1 μL of 4-methyl-2-pentanol was added as an internal standard at the concentration of 401 μg/μL. After the equilibrium of 60 °C for 20 min, a stainless steel needle, housing the SPME fiber, was placed through the hole to expose the fiber at the position of 1 cm over the liquid surface for 40 min. The vials were sealed tightly with screw caps fitted with a Teflon/silicon septum. Vials were continuously swirled during SPME exposure with an agitation speed of 100 rpm.

### 3.4. Aroma Extraction of EVOO by SAFE

The method was in accordance with Usami et al. with minor modification [39]. Fifty milliliters of EVOO was transferred into 250 mL Teflon bottle, then 150 mL of dichloromethane and 1 μL of 4-methyl-2-pentanol was added as an internal standard at the concentration of 401 μg/μL. The bottle was placed in a shaker for 8h at 4 °C and 180r/min. The volatile compounds were separated from the solvent extracts using SAFE [17]. The filtrate was vacuum distilled using SAFE apparatus as previously described [40].

### 3.5. Gas Chromatography-Olfactometry-Mass Spectrometry (GC-MS/O) Analysis

The method was in accordance with Nuzzi et al. with minor modification [41]. The qualitative and quantitative analyses of the volatile compounds were conducted using Agilent 7890A gas chromatograph coupled with an Agilent model 7000B series mass spectrometer (GC-MS) and desorbed for 7 min in a split/splitless GC injection port, which was equipped with an inlet linear specific for SPME use (Agilent Technologies, Wilmington, DE, USA). The GC-MS was equipped with a sniffer 9000 Olfactometer (Brechbühler, Switzerland). The volatiles were separated on DB-5 and DB-Wax (30 m × 0.25 mm i.d. × 0.25 μm, J&W Scientific), a type of fused silica capillary columns.

The oven temperature was initially at 40 °C, held for 3 min, ramped at 5 °C/min to 200 °C, then ramped at 10 °C /min to 230 °C and held for 3 min, then baked at 250 °C for 3 min. The injection port and ionizing source were kept at 250 and 230 °C, respectively; the carrier gas was helium at 1.2 mL/min. The injector mode was splitless. Electron-impact mass spectra were generated at 70 eV, with an m/z scan range from 35 to 350 amu. Compounds were identified according to NIST 14.0 mass spectra libraries installed in the GC-MS equipment.

A sniffing port (Sniffer 9000) coupled to a GC-MS instrument was used for odor-active compound characterization. At the exit of the capillary column, the effluents were split 1:1 (by volume) into a sniffing port and a MS detector by employing the Agilent capillary flow technology; the transfer line to the GC/O sniffing port was held at 280 °C. GC/O was performed by three experienced panelists.

### 3.6. Aroma Extract Dilution Analysis (AEDA)

The highest sample concentration after SAFE was assigned with a FD factor of 1. The volatile components were stepwise diluted at the ratio of 1: 2 with dichloromethane, and aliquots of the dilutions (1 μL) were subjected to analysis. The process was stopped when aromas ceased to be detected by the evaluators. The result was expressed as the FD factor, which was the ratio of initial and final concentration of the odorant in the sample.

### 3.7. Identification of Volatile Aroma Compounds

The method was in accordance with Xu et al. with minor modification [42]. The chemical identification was performed using a mass spectrum database, the linear retention index (*LRI*), and aromatic characteristics by sniffing. Some important aroma compounds were identified by comparison with standard compounds. *LRI* was calculated using normal alkane series and compared with the references. Mass spectra identification was performed based on the NIST 2.0 mass spectra libraries. The formula for the calculation of *LRI* was (1).

(1) $$LRI=100\times \left(n+\frac{t_{a}{-t}_{n}}{t_{n+1}{-t}_{n}}\right)$$

where “n” represents the number of carbon atoms of n-paraffins; “*t*_n_” represents the retention time of n-paraffins *C*_n_, “*t*_n+1_” represents the retention time of n-paraffins *C*_n+1_, “*t*_a_” represents the retention of an unknown compound in the sample time (to be satisfied “*t*_a_” is between “*t*_n_” and “*t*_n+1_”).

### 3.8. Quantification of Volatile Aroma Compounds

SPME was used to extract the volatile aroma components in olive oil for quantification analysis. At the same time, two quantitative methods were used, including an internal standard method for total aroma compounds and an external standard method in SIM mode for the key aroma compounds. In the internal standard method, 4-methyl-2-pentanol (401 μg/μL in hexane) was added as an internal standard to the sample to calculate the target compound concentration.

(2) $$CX=\frac{AX}{AIS}CIS$$

where “*C*_IS_” represents the concentration of internal standard; “*A*_IS_” represents the peak area of the internal standard; “*C*_X_” represents the concentration of the target compound; “*A*_X_” represents the peak area of the target compound.

Hexanal, (*E*)-2-hexenal, (*Z*)-3-hexenyl acetate, (*E*)-2-heptenal, 6-methyl-5-hepten-2-one, (*Z*)-3-hexenol, and acetic acid were quantified using SIM mass spectrometry by standard curve method. The solutions of the mixture of 4-methyl-2-pentanol and reference compounds at different concentrations were prepared and analyzed by GC-MS. The standard curves were prepared by plotting the ratio of the peak areas of the reference compound relative to 4-methyl-2-pentanol against their concentration ratio.

### 3.9. Calculation of OAV

OAV was calculated using the following equation:

(3) $$OAV=\frac{C_{i}}{OT_{i}}$$

where *C_i_* is the concentration of the compound in the watermelon juice and *OT_i_* is its odor threshold. Compounds with OAV equal to or greater than 1 actually contribute to aroma as an odor-active compound because they are above their odor threshold, whereas those with OAV smaller than 1 may not.

### 3.10. Statistical Analysis

Analysis of variance (ANOVA) were carried out by using the software SAS (SAS Institute Inc., Cary, NC, USA). The ANOVA test was performed for all experimental runs, to determine the significance at 95% confidence interval. All experiments were performed in triplicate. The cluster analysis was applied to the concentration of volatile compounds data, and it was conducted by using the software of Morpheus online (https://software.broadinstitute.org/morpheus/). In this process, hierarchical clustering was selected, “Euclidean distance” and “Average” were applied to calculate the distance of samples and groups.

## 4. Conclusions

Eighty-nine compounds were screened by SPME/SAFE-GC-MS/O with chromatographic columns of DB-Wax and DB-5 in 15 brands of EVOOs. Eighty compounds were identified by SPME-GC-MS/O and 54 by SAFE-GC-MS/O method. Forty-four compounds were detected by both methods. Undecanol, (*Z*)-4-decenal, (*E*)-2-dodecenal, and 2-nonanone were found in EVOOs for the first time only by SAFE. 2-Nonanone was the key aroma compounds identified by AEDA. SPME had better extraction efficiency to aroma compounds in EVOOs, while SAFE could extract the most effective components (seven key aroma compounds). Hence, it is feasible and advantageous that SAFE is applied to the aroma extraction of EVOOs.

Eight classes of flavor compounds were identified in the 15 brands of EVOOs, including 17 alcohols, 22 aldehydes, 9 ketones, 4 acids, 14 esters, 5 aromatics, 12 alkene, and 6 others. Eleven compounds were identified as the key aroma compounds in alternative brands of EVOOs by SAFE-AEDA. Key aroma compounds authenticated by AEDA had some differences from those by OAVs. The aroma compounds of Spain EVOOs were similar, and those of some of samples from Italy and Greece were alike.

## Figures and Tables

**Table 1:** All the flavor compounds identified in 15 EVOOs by SPME and SAFE.

Compounds	CAS	LRI		Odor Property	ID ^1^	Extraction Method	Brands of EVOOs ^2^
		DB-Wax	DB-5				
***Alcohols***							
(*Z*)-2-Pentenol	1576-95-0	1157		green, plastic	LRI, MS	SPME	11
1-Pentene-3-ol	616-25-1	1157	686	fruity, nut	LRI, MS, O	SPME/SAFE	5, 10, 12-15/6, 8-10, 14, 15
Pentanol	71-41-0	1206		balsamic	LRI, MS	SPME	3, 6, 7
Eucalyptol	470-82-6	1206		mint, sweet	LRI, MS	SPME	15
Hexanol	111-27-3	1345	868	resin, flower, green	LRI, MS	SPME/SAFE	1-4, 6-9, 11-14/1-15
(*E*)-3-Hexenol	928-97-2	1376		moss, fresh	LRI, MS, O	SAFE	1-15
(*E*)-2-Hexenol	928-95-0	1397	866	green, leaf, walnut	LRI, MS	SPME/SAFE	1, 9, 12/1, 5-15
2-Ethyl hexanol	104-76-7	1481	1030	rose, green	LRI, MS	SPME/SAFE	4-6, 13, 14/4-15
Linalool	78-70-6	1538	1099	flower, lavender	LRI, MS	SPME/SAFE	5, 13/1-6, 9, 10, 13, 15
Octanol	111-87-5	1549		chemical, metal, burnt	LRI, MS	SPME/SAFE	2, 3, 8/1-3, 9, 10, 12-15
Nonanol	143-08-8	1556	1172	fat, green	LRI, MS	SPME	2, 4, 7-10, 12-15
Undecanol	112-42-5	1653		mandarin	LRI, MS	SAFE	3, 6, 9, 10, 12-15
Benzyl alcohol	100-51-6	1863	1038	sweet, flower	LRI, MS	SPME/SAFE	4, 7, 8, 12, 13, 15/1-5, 7-15
Phenylethyl alcohol	60-12-8	1899	1115	honey, spice, rose, lilac	LRI, MS	SPME/SAFE	1-15/1-15
(*Z*)-2-Hexenol	928-94-9		866	leaf, green, wine, fruit	LRI, MS	SPME	8, 15
(*Z*)-3-Hexenol	928-96-1	1382	855	grass	LRI, MS, O	SPME/SAFE	1-15/1-15
1-Octen-3-ol	3391-86-4		979	mushroom	LRI, MS	SPME	2, 5, 6, 12, 15
***Aldehydes***							
2-Methylbutyraldehyde	96-17-3	905		cocoa, almond	LRI, MS	SPME	13
Hexanal	66-25-1	1081	801	grass, tallow, fat	LRI, MS, O, STD	SPME/SAFE	1-15/1-15
(*E*)-2-Pentenal	1576-87-0	1126		strawberry, fruit, tom	LRI, MS, O	SPME	3-5, 12, 13, 15
(*E*)-2-Hexenal	6728-26-3	1214	852	apple, green	LRI, MS, O, STD	SPME/SAFE	1-15/1-15
(*Z*)-2-Hexenal	505-57-7		848	fat, rancid	LRI, MS, O	SPME	7-12, 14, 15
Octanal	124-13-0	1285	1003	fat, soap, lemon, green	LRI, MS	SAFE	1-15/1-3, 5-15
(*E*)-2-Heptenal	18829-55-5	1319	956	soap, fat, almond	LRI, MS, O, STD	SPME/SAFE	1-3, 5-13/1-3, 5-15
2,6-Dimethyl-5-heptenal	106-72-9	1361		fruit, green, melon	LRI, MS	SPME	3
Nonanal	124-19-6	1388	1105	fat, citrus, green	LRI, MS, O	SPME/SAFE	1-15/1-15
(*E*)-2-Octenal	2548-87-0	1425	1059	green, nut, fat	LRI, MS, O	SPME/SAFE	1-15/1-3, 5-8, 10-12
(*E,E*)-2,4-Heptadienal	4313-03-5	1456	998	nut, fat	LRI, MS, O	SPME/SAFE	1-15/1-15
2-Furanaldehyde	98-01-1	1457		bread, almond, sweet	LRI, MS	SPME	3
Benzaldehyde	100-52-7	1521		almond, burnt sugar	LRI, MS	SPME	1-12/15
Phenylacetaldehyde	122-78-1		1044	hawthorn, honey, sweet	LRI, MS	SPME	1, 4, 6, 10, 14
(*E*)-2-Nonenal	18829-56-6	1530	1161	cucumber, fat, green	LRI, MS, O, STD	SPME/SAFE	1-3, 5-15/1-3, 5-15
(*Z*)-2-Decenal	2497-25-8		1263	tallow	LRI, MS	SPME	10
(*Z*)-4-Decenal	21662-09-9	1605		green, must	LRI, MS	SAFE	6, 11, 14
(*E*)-2-Decenal	3913-81-3	1640	1264	tallow	LRI, MS	SPME/SAFE	1-9, 11-15/1-15
2-Undecenal	2463-77-6	1748	1369	soap, fat, green	LRI, MS	SPME/SAFE	3/1, 6, 8, 14
(*E*)-2-Dodecenal	20407-84-5	1749		green, fat, sweet	LRI, MS	SAFE	3, 7
(*E,E*)-2,4-Decadienal	25152-84-5	1803	1321	fried, wax, fat	LRI, MS	SPME/SAFE	1-3, 5-7, 9-13/1-3, 5-11, 13, 14
Heptanal	111-71-7	1181	902	fat, citrus, rancid	LRI, MS	SPME/SAFE	1-3, 5, 11, 13, 15/1, 3, 6, 7, 13, 15
***Ketones***							
2-Pentanone	107-87-9	977		ether, fruit	LRI, MS	SPME	1-6
3-Pentanone	96-22-0	980		ether	LRI, MS	SPME	4-8, 10, 13-15
1-Penten-3-one	1629-58-9	1017	<800	fish, pungent	LRI, MS, O	SPME	4, 13-15
2-Octanone	111-13-7	1281		soap, gasoline	LRI, MS, O, STD	SPME/SAFE	1, 3, 12, 13/3
1-Octen-3-one	4312-99-6	1302		mushroom, metal	O, STD	SAFE	1-15
6-Methyl-5-hepten-2-one	110-93-0	1331	987	pepper, mushroom, rubber	LRI, MS, O, STD	SPME/SAFE	1-15/1-15
2-Nonanone	821-55-6	1404		hot milk, soap	O, STD	SAFE	3, 6-8, 13-15
3,5-Octadien-2-one	38284-27-4	1511	1072	fruit, fat, mushroom	LRI, MS	SPME/SAFE	1, 5-9, 11-15/1-10, 12-15
Acetophenone	98-86-2	1649		must, flower, almond	LRI, MS	SPME	2, 5
***Acids***							
Acetic acid	64-19-7	1435		sour	LRI, MS, O, STD	SPME/SAFE	1, 4, 5, 8, 11, 13, 15/1-13, 15
Propionic acid	79-09-4	1524		pungent, rancid, soy	LRI, MS, O	SPME/SAFE	1-3, 5, 6, 9, 11-15/2, 3, 6-10, 12, 14, 15
Hexanoic acid	142-62-1	1832	990	sweat	LRI, MS, O	SPME/SAFE	1, 7, 11, 13, 15/2, 3, 7, 9, 11, 13, 15
Nonanoic acid	112-05-0		1273	green, fat	LRI, MS	SPME/SAFE	1, 2, 5, 7, 9, 10, 12, 13, 15/1, 6, 8-10, 13-15
***Esters***							
Ethyl acetate	141-78-6	<900		pineapple	LRI, MS	SPME	2-4, 8, 10, 13
Methyl hexanoate	106-70-7		922	fruit, fresh, sweet	LRI, MS, O	SPME	3, 6
Ethyl Hexanoate	123-66-0	1231		apple peel, fruit	LRI, MS	SPME/SAFE	2, 3, 6/2, 3, 5
Hexyl acetate	142-92-7	1268	1013	fruit, herb	LRI, MS	SPME/SAFE	1-15/1-15
(*Z*)-3-Hexenyl acetate	3681-71-8	1311	1007	green, banana	LRI, MS, STD	SPME/SAFE	1-15/1-15
Linalyl acetate	115-95-7	1544		sweet, fruit	LRI, MS	SPME	5
Methyl benzoate	93-58-3	1613	1098	prune, lettuce, herb, sweet	LRI, MS, O	SPME/SAFE	1-15/1-7, 9, 10, 12-14
γ-Butyrolactone	96-48-0	1626		caramel, sweet	LRI, MS	SPME	2, 3
Methyl octanoate	111-11-5		1123	orange	LRI, MS	SPME	2, 3, 6
Ethyl benzoate	93-89-0	1661	1172	chamomile, flower, celery, fruit	LRI, MS	SPME/SAFE	1-7, 9, 11/2-7, 9
γ-Hexanolide	695-06-7	1701		coumarin, sweet	LRI, MS	SPME	7
Methyl salicylate	119-36-8	1769	1199	peppermint	LRI, MS	SPME/SAFE	4-10, 12-15/2-5, 7, 9-11, 13-15
Nonyl ethanoate	143-13-5		1308	sweet, fruit	LRI, MS	SPME	7
Butyl acetate	123-86-4		815	pear	LRI, MS	SAFE	1, 4, 5
***Aromatics***							
Methylbenzene	108-88-3	1041	<800	paint	LRI, MS, O	SPME/SAFE	1, 2, 4-8, 10, 12-15/1, 2, 5, 6
1,2-Dimethylbenzene	95-47-6	1138	870	geranium	LRI, MS, O	SPME/SAFE	3, 5-8, 12, 13, 15/1-3, 5-15
1,3-Dimethylbenzene	108-38-3	1182	861	plastic	LRI, MS, O	SPME/SAFE	1-3, 5-8, 10-15/1, 2, 5, 7, 9, 10
1,2,4-Trimethylbenzene	95-63-6	1277	969	plastic	LRI, MS	SAFE	1-3, 5, 6, 8-15/1, 5, 6, 8, 10, 12-15
1,2,4,5-Tetramethylbenzene	95-93-2	1436		rancid, sweet	LRI, MS	SPME	1, 7
***Terpenes***							
Cinene	138-86-3		1029	lemon, orange	LRI, MS	SPME	1, 2, 7-9, 15
(+)-Dipentene	5989-27-5	1198	1032	citrus, mint	LRI, MS	SPME/SAFE	3, 8, 10, 11, 13, 15/1-3, 6-11, 13, 15
3-Carene	13466-78-9	1233	1049	lemon, resin	LRI, MS, O	SPME	1-4, 6-12, 14, 15
(E)-Ocimene	3779-61-1	1235		sweet, herb	LRI, MS	SPME	7
(*Z*)-3,7-Dimethyl-1,3,6-octatriene	3338-55-4	1249	1054	citrus, herb, flower	LRI, MS	SPME/SAFE	1-13, 15/1-4, 9, 11, 15
Perillen	539-52-6		1116	wood	LRI, MS	SPME	2, 3, 6, 7, 9, 13, 15
Ethenylbenzene	100-42-5	1252	892	balsamic, gasoline	LRI, MS	SPME/SAFE	1-3, 5, 6, 11/1-6, 11-15
(-)-α-Cubebene	17699-14-8	1495		herb, wax	LRI, MS	SPME	8, 15
(-)-α-Copaene	3856-25-5	1495	1387	wood, spice	LRI, MS	SPME/SAFE	1-15/1-15
4-Methoxystyrene	637-69-4	1675		sweet	LRI, MS	SPME	5
(+)-Valencene	4630-07-3	1717		green, oil	LRI, MS	SPME	5
α-Farnesene	502-61-4	1743	1512	wood, sweet	LRI, MS, O	SPME/SAFE	1-15/1-15
***Others***							
2-Pentylfuran	3777-69-3	1231		green bean, butter	LRI, MS	SPME	1, 3, 5, 11
Benzyl methyl ether	538-86-3	1386	988	metal	LRI, MS, O	SPME/SAFE	2-6, 10/2, 6, 10
Dimethyl sulfoxide	67-68-5	1568		garlic	LRI, MS	SPME/SAFE	1-7, 11/2-5, 7, 11
Naphthalene	91-20-3	1734	1189	tar	LRI, MS	SPME/SAFE	5,7, 11-15/2, 5-14
4-Ethylphenol	123-07-9	2159		phenol, spice	LRI, MS	SPME/SAFE	3-6, 11/1-3, 5, 7, 8, 10, 11, 13
4-Allylanisole	140-67-0		1275	licorice, anise	LRI, MS	SPME	13

^1^ Each compound was identified by comparing it with an authentic standard based on the following criteria: (i) matching linear retention index on the same column; (ii) mass spectrum on NIST 14.0 database; (iii) description of its odor description; (iv) injection of reference standards. ^2^ 1-PL, 2-BDS, 3-OL, 4-BLN, 5-YGY, 6-OLWL, 7-OS, 8-ALCF, 9-MNN, 10-AN, 11-XBK, 12-DMDN, 13-MSWN, 14-AGL, 15-DNLE.

**Table 2:** Key aroma compounds identified in 15 EVOOs by FD.

Compounds	FD														
	PL	BDS	OL	BLN	YGY	OLWL	OS	ALCF	MNN	AN	XBK	DMDN	MSWN	AGL	DNLE
Hexanal	32	32	16	64	16	16	64	32	32	64	32	32	64	32	32
(*E*)-2-Hexenal	64	64	64	16	64	16	8	8	16	16	64	8	4	4	2
2-Octanone	4	2	4	-	4	2	4	16	2	4	0	4	8	4	4
1-Octen-3-one	8	8	4	4	16	8	32	16	16	8	4	32	16	16	16
(*E*)-2-Heptenal	-	2	2	4	4	4	8	4	8	8	4	8	4	16	8
(*E*)-3-Hexenol	8	4	4	4	4	4	8	0	2	2	0	0	4	4	4
Nonanal	-	-	-	-	-	2	2	2	0	4	8	-	4	2	0
2-Nonanone	-	-	0	-	-	4	4	0	-	-	-	-	2	0	2
Acetic acid	4	2	4	-	4	-	4	16	2	2	2	4	0	-	-
(*E*)-2-Nonenal	16	-	2	0	2	4	2	2	2	4	4	2	4	2	0
Methyl benzoate	-	2	-	-	8	4	-	-	-	2	4	2	-	-	-

**Table 3:** Standard curve, characteristic ions, and concentration of key aroma compounds in 15 EVOOs by external standard.

Compounds	Ion Selection (m/z)	Standard Curve	*R* ^2^	Concentration (mg/L)														
				PL	BDS	OL	BLN	YGY	OLWL	OS	ALCF	MNN	AN	XBK	DMDN	MSWN	AGL	DNLE
Hexanal	56.2, 72.2, 82.2	y = 0.1546x	0.9950	1.06 ± 0.17	0.92 ± 0.12	1.88 ± 0.39	0.64 ± 0.14	2.16 ± 0.22	1.23 ± 0.09	4.27 ± 0.03	1.69 ± 0.09	1.72 ± 0.03	1.84 ± 0.03	4.31 ± 0.10	1.37 ± 0.07	1.90 ± 0.04	1.76 ± 0.18	2.00 ± 0.11
(*E*)-2-Hexenal	69.2, 83.2, 98.2	y = 0.5272x	0.9953	0.30 ± 0.01	1.01 ± 0.14	0.33 ± 0.07	0.69 ± 0.11	1.58 ± 0.03	1.52 ± 0.18	4.61 ± 0.77	3.05 ± 0.12	2.67 ± 0.14	3.81 ± 0.12	3.92 ± 0.00	3.25 ± 0.46	1.66 ± 0.01	3.87 ± 0.17	6.99 ± 0.35
(*Z*)-3-Hexenyl acetate	67.2, 82.2	y = 0.8552x	0.9940	0.86 ± 0.09	2.22 ± 0.25	0.72 ± 0.16	2.32 ± 0.29	1.01 ± 0.02	1.87 ± 0.20	3.00 ± 0.65	1.19 ± 0.01	1.92 ± 0.19	2.22 ± 0.23	1.80 ± 0.01	2.10 ± 0.42	1.93 ± 0.09	3.70 ± 0.19	3.96 ± 0.08
(*E*)-3-Hexenol	55.2, 67.2, 82.2	y = 0.3685x	0.9974	0.72 ± 0.01	1.63 ± 0.24	0.63 ± 0.10	1.60 ± 0.16	1.32 ± 0.05	1.41 ± 0.09	1.69 ± 0.33	1.09 ± 0.00	1.19 ± 0.05	1.20 ± 0.15	0.83 ± 0.05	1.03 ± 0.10	0.74 ± 0.03	0.64 ± 0.91	0.83 ± 0.01
Acetic acid	60.2	y = 0.1964x	0.9970	2.32 ± 0.18	3.94 ± 0.58	2.21 ± 0.33	4.59 ± 0.38	2.14 ± 0.09	0.64 ± 0.05	3.05 ± 0.50	2.38 ± 0.10	3.07 ± 0.08	1.54 ± 0.18	0.82 ± 0.03	0.81 ± 0.04	0.86 ± 0.02	0.55 ± 0.03	1.21 ± 0.04
(*E*)-2-Heptenal	55.2, 83.2, 112.2	y = 8.4945x	0.9948	0.30 ± 0.01	0.13 ± 0.01	0.48 ± 0.03	0.06 ± 0.01	0.24 ± 0.02	0.36 ± 0.01	0.44 ± 0.04	0.26 ± 0.02	0.39 ± 0.01	0.31 ± 0.01	0.43 ± 0.03	0.43 ± 0.03	0.31 ± 0.01	0.14 ± 0.02	0.26 ± 0.01
6-Methyl-5-hepten-2-one	69.2, 108.2, 126.2	y = 30.406x	0.9982	0.04 ± 0.00	0.04 ± 0.00	0.07 ± 0.01	0.02 ± 0.00	0.09 ± 0.01	0.06 ± 0.00	0.11 ± 0.01	0.06 ± 0.00	0.06 ± 0.00	0.08 ± 0.00	0.03 ± 0.00	0.08 ± 0.01	0.06 ± 0.00	0.03 ± 0.00	0.07 ± 0.00

**Table 4:** Odor activity values (OAVs) of key aroma compounds identified in 15 EVOOs.

Compounds	Threshold [19]	OAV														
		PL	BDS	OL	BLN	YGY	OLWL	OS	ALCF	MNN	AN	XBK	DMDN	MSWN	AGL	DNLE
Hexanal	80	12	11	22	7	25	14	49	19	20	21	50	16	22	20	23
(*E*)-2-Hexenal	420	1	2	1	2	3	3	10	7	6	8	9	7	4	8	15
(*Z*)-3-Hexenyl acetate	6.9	115	296	96	309	135	249	400	159	256	296	240	280	257	493	528
(*E*)-3-Hexenol	1100	<1	1	<1	1	1	1	1	1	1	1	1	1	1	1	1
Acetic acid	500	4	7	4	8	4	1	6	4	6	3	2	1	2	1	2
(*E*)-2-Heptenal	5	55	24	88	11	44	66	81	48	72	57	79	79	57	26	48
6-Methyl-5-hepten-2-one	1000	<1	<1	<1	<1	<1	<1	<1	<1	<1	<1	<1	<1	<1	<1	<1

**Table 5:** The detection frequency of compounds based on the kinds of EVOOs and compound groups.

	Number (EVOOs of Spain)					*Subtotal*	Number (EVOOs of Italy)					*Subtotal*	Number (EVOOs of Greece)					*Subtotal*
	PL	BDS	OL	BLN	YGY		OLWL	OS	ALCF	MNN	AN		XBK	DMDN	MSWN	AGL	DNLE	
**Alcohols**	5	7	9	8	10	**39**	10	9	11	12	12	**54**	8	12	12	11	15	**58**
**Aldehydes**	13	12	17	10	13	**65**	13	13	12	11	14	**63**	14	13	13	12	13	**65**
**Ketones**	5	5	6	6	6	**28**	6	5	5	3	4	**23**	3	4	7	6	6	**26**
**Acids**	4	4	3	1	3	**15**	3	4	3	4	3	**17**	3	3	4	2	4	**16**
**Esters**	5	9	10	7	8	**39**	8	7	5	5	5	**30**	5	4	5	4	3	**21**
**Aromatics**	4	4	3	1	4	**16**	4	3	4	3	4	**18**	3	4	4	4	4	**19**
**Terpenes**	7	8	7	5	4	**31**	7	8	7	7	5	**34**	6	5	6	4	9	**30**
**Others**	3	4	4	3	5	**19**	3	3	2	1	3	**12**	4	1	3	1	0	**9**
***Subtotal***	**46**	**53**	**59**	**41**	**53**		**54**	**52**	**49**	**46**	**50**		**46**	**46**	**54**	**44**	**54**	
